# Hormones, Age, and Erectile Dysfunction: Should Routine Testing Be Part of the Initial Evaluation?

**DOI:** 10.3390/diagnostics15030294

**Published:** 2025-01-27

**Authors:** Daniel Porav-Hodade, Raul Dumitru Gherasim, Irina Bianca Kosovski, Toader Septimiu Voidazan, Nicolae Crisan, Petrut Bogdan, Radu Galis, Bogdan Ovidiu Feciche, Mártha Orsolya Katalin Ilona, Ciprian Todea-Moga

**Affiliations:** 1 Department of Urology, George Emil Palade University of Medicine, Pharmacy, Science, and Technology of Târgu Mureș, 540139 Târgu Mureș, Romania; daniel.porav-hodade@umfst.ro (D.P.-H.); orsolya.martha@umfst.ro (M.O.K.I.); ciprian.todea@umfst.ro (C.T.-M.); 2 Department of Urology, Clinical County Hospital Mures, 540136 Târgu Mures, Romania; 3 Antares Clinic, 610006 Piatra Neamt, Romania; 4 Department of Pathophysiology, George Emil Palade University of Medicine, Pharmacy, Science and Technology of Târgu Mureș, 540139 Târgu Mureș, Romania; bianca.kosovski@umfst.ro; 5 Department of Laboratory, Clinical County Hospital Mures, 540136 Târgu Mures, Romania; 6 Department of Epidemiology, George Emil Palade University of Medicine, Pharmacy, Science, and Technology of Târgu Mureș, 540139 Târgu Mureș, Romania; septimiu.voidazan@umfst.ro; 7 Department of Urology, Iului Hatieganu University of Medicine and Pharmacy, 400347 Cluj-Napoca, Romania; nicolae.crisan@umfcluj.ro (N.C.); bogdan.petrut@gmail.com (P.B.); 8 Department of Medical Sciences, Faculty of Medicine and Pharmacy, University of Oradea, 410087 Oradea, Romania; 9 Department of Urology, Faculty of Medicine and Pharmacy, University of Oradea, 410087 Oradea, Romania; feciche.bogdanovidiu@didactic.uoradea.ro; 10 Department of Urology, Emergency County Hospital Oradea, 410169 Oradea, Romania

**Keywords:** age, erectile dysfunction, total testosterone, free testosterone, FSH, LH, estradiol, SHBG, prolactin

## Abstract

**Background/Objectives**: The aim of this study was to investigate the relationship between age, the severity of erectile dysfunction (ED), and the various hormones that may influence erectile function. **Methods**: A multicenter cross-sectional study was conducted between January 2015 and December 2023. The study assessed age, sexual function using the IIEF-15 questionnaire, and the levels of total testosterone (TT), free testosterone (FT), FSH, LH, estradiol, prolactin (PRL), and SHBG. **Results**: A total of 411 patients were included in the study. The mean age of the patients was 63.19 years. The vast majority (91.73%) exhibited some degree of ED. The severity of ED increases with age, ranging from 56.26 years for patients without ED to 73.12 years for those with severe ED. A statistically significant negative correlation was observed between IIEF and age, while a positive correlation was observed between IIEF and serum levels of TT and FT (*p* < 0.05). Age was significantly correlated with all evaluated hormones (*p* < 0.01), except estradiol and prolactin. Total testosterone levels progressively decreased with the increase in the severity of erectile dysfunction, from a median of 7.05 ng/mL in patients with normal erectile function to 3.56 ng/mL in those with severe symptoms, remaining above the normal minimum threshold across all groups, whereas free testosterone (FT) levels also declined progressively. All erectile dysfunction groups had median FT levels below the normal minimum threshold. FSH, LH, and SHBG showed an increase with each progressive severity of erectile dysfunction. The multivariate linear regression revealed that IIEF scores are significantly associated with age, TT, and FT levels, while FSH did not present a statistically significant association in this model. **Conclusions**: Age shows a significant statistical correlation with both the severity of erectile dysfunction and the levels of total testosterone, free testosterone, LH, FSH, and SHBG. Total and free testosterone levels are significantly associated with the severity of erectile dysfunction, with free testosterone median values remaining above the normal minimum threshold in all patients with erectile dysfunction. Therefore, free testosterone should be considered a routine test, alongside total testosterone. In contrast, LH, estradiol, SHBG, and prolactin do not demonstrate any statistical correlation with erectile dysfunction and should not be recommended as routine investigations.

## 1. Introduction

Erectile dysfunction (ED) is the persistent inability to achieve and maintain an erection sufficient to permit satisfactory sexual performance [1]. This will have a significant impact, greatly affecting the quality of life for both the patient and their partner [2,3].

The quality of studies on ED varies widely due to the use of diverse assessment tools, population biases, and the varying interpretations of the findings [4]. Among these studies, the Massachusetts Male Aging Study (MMAS) stands out as a landmark population-based longitudinal study [5]. It provided foundational insights into the prevalence, risk factors, and progression of ED. The MMAS reported that the combined prevalence of minimal, moderate, and complete ED was 52%, with the prevalence of severe erectile dysfunction tripling between the ages of 40 and 70 years.

Recent data from the 2021 National Survey of Sexual Wellbeing [6] highlighted that the prevalence of ED in the United States, based on IIEF-5 scores, was 24.2%. Age was a significant factor, with 52.2% of men aged 75 years and older and 48.0% of those aged 65–74 years meeting the diagnostic criteria for ED.

Why is erectile function such a significant topic? For many men, sexual vitality is closely tied to their sense of well-being and fulfillment in life [7]. Sexually active men tend to be happier [8], experience lower rates of depression [9,10], and enjoy a higher quality of life overall [11]. However, only a small number of patients with erectile dysfunction receive treatment [12].

The onset of ED is influenced by a combination of genetic predisposition [13,14] and a multifaceted interaction, including anatomical, vascular, neurological, psychological factors, and lifestyle factors [15]. In addition to this, hormonal factors play a critical role in the development and progression of ED [16], even though endocrinopathies are among the rarest causes [17,18]. Despite their importance, hormonal factors are frequently overlooked in clinical assessments, leading to missed opportunities for targeted interventions, such as testosterone replacement therapy or addressing endocrine disorders [19]. This highlights the need for a comprehensive evaluation of hormonal health in patients presenting with ED.

One of the significant gaps in the hormonal evaluation of ED is the lack of standardization in testing and interpretation [20,21]. Although total testosterone is widely tested, there is increasing evidence suggesting that measuring free testosterone may be more useful in diagnosing hypogonadism and its impact on erectile function. However, free testosterone testing is not as commonly conducted in clinical practice, and its inclusion in routine evaluations remains inconsistent. Additionally, there is limited consensus on the necessity of testing other hormones, such as FSH, LH, prolactin, SHBG, and estradiol.

Testosterone, both total (TT) and free (FT), is essential for sexual health and erectile function [22]. TT helps regulate libido and nitric oxide production in penile tissues [23,24]. Low TT levels are often associated with reduced libido, nocturnal erection, and overall poor sexual performance [25,26].

Free testosterone directly activates androgen receptors in the corpora cavernosa, maintaining erectile function [27].

Elevated SHBG reduces FT availability, and men with both low FT and high SHBG levels face the highest risk of erectile dysfunction [28]. Low SHBG is linked to metabolic disorders such as obesity and insulin resistance, which are independent risk factors for ED [29].

FSH and LH regulate testosterone production and spermatogenesis, and their levels can be correlated with ED [30,31]. Elevated LH with low testosterone indicates primary hypogonadism [32,33], while low LH suggests secondary hypogonadism, both leading to testosterone deficiency and ED [34,35].

Estradiol, although essential for male sexual health, can disrupt erectile function when levels are imbalanced [36]. High estradiol, often due to obesity, suppresses testosterone and impairs vascular function [37,38].

Hyperprolactinemia can cause hypogonadism by interfering with GnRH secretion, but not all individuals with high prolactin levels experience ED [39,40]. Abnormally low prolactin may also contribute to ED by disrupting dopaminergic pathways regulating sexual desire [41].

The reasons that triggered this study stem from the increasing recognition of ED as a common condition that significantly impacts quality of life. There is a general lack of comprehensive guidelines for hormonal testing in ED, resulting in variability in clinical practice. This motivated us to investigate the current state of hormonal evaluation and contribute to the ongoing discussion about enhancing the diagnostic process for ED. This study aims to address these knowledge gaps, standardize hormonal evaluations, and enhance the overall approach to diagnosing and treating ED. Specifically, it seeks to investigate the relationship between age, the severity of erectile dysfunction, and various hormones that may influence erectile function.

## 2. Materials and Methods

### 2.1. Participant Selection

This multicenter, retrospective, cross-sectional study involved patients from centers in Romania between January 2015 and December 2023. The period from January 2020 to December 2021 was excluded due to inconsistent and uneven data caused by the COVID-19 pandemic. Data collection for this study was conducted through local awareness campaigns led by general practitioners, focusing on the evaluation of sexual health issues in relation to potential hormonal changes in male patients. The general practitioners disseminated information to their patients, raising awareness about the study and its objectives.

#### 2.1.1. Assessment of the Severity of Erectile Dysfunction

The severity of erectile dysfunction was assessed using the IIEF-15 questionnaire. The International Index of Erectile Function (IIEF-15) is a well-established and validated tool for evaluating sexual function. It includes 15 questions that comprehensively examine five domains: erectile function, orgasmic function, sexual desire, satisfaction during intercourse, and overall sexual satisfaction. Patients were categorized into distinct groups according to the severity of sexual dysfunction: no dysfunction (61–75 points), mild dysfunction (41–60 points), moderate dysfunction (21–40 points), and severe dysfunction (≤20 points).

#### 2.1.2. Hormonal Evaluations

Venous blood samples were collected in the morning following an 8–10-h fast. BD Vacutainers^®^ (Becton, Dickinson and Company, Franklin Lakes, NJ, USA) contain-ing a clot activator and gel serum separator were used. After 30 min, serum was isolated by centrifugation and aliquoted into 5.0 mL Eppendorf® tubes (Eppendorf, Hamburg, Germany). All samples were analyzed using ARCHITECT i1000 analyzer (Abbot Diagnostics, Berkshire, UK) based on the chemiluminescence immunoassay method. The following serum analyte concentrations were measured: FSH (mIU/mL; reference range: 1.4–15.4), LH (mIU/mL; reference range: 1.8–8.6), total testosterone (TT, nmol/L; reference range: 10.40–34.67), estradiol (pmol/L; reference range: 27.90–156.40), prolactin (PRL, ng/mLμg/L; reference range: 8–14), and sex hormone-binding globulin (SHBG, nmol/L; reference range: 10–57). The Vermeulen formula was used to calculate free testosterone (FT): FT = ((TT − N − SHBG + √ ((N + SHBG − TT) 2 + 4 × N × TT))/2N)) × (100/TT), where N = 0.5217 × albumin concentration + 1 (pmol/L, reference range: 27.73–74.54) [13].

#### 2.1.3. Inclusion Criteria

The study included patients who completed the IIEF-15 questionnaire and agreed to blood sampling for the hormonal tests mentioned before. There was no age limit. All patients included in the study were treatment-naive for benign prostatic hyperplasia (BPH), erectile dysfunction, or hormone therapy.

#### 2.1.4. Exclusion Criteria

Patients who had undergone urological surgeries (such as TURP, urethrotomies, radical prostatectomy, radical cystectomy, or unilateral/bilateral orchiectomy, penectomy) and those who had general surgery procedures (such as rectal amputation or surgeries involving the sigmoid colon) were excluded, even if they met the study’s inclusion criteria. Additionally, patients who did not fully complete the IIEF questionnaire, those whose laboratory results were collected outside the specified time window, or those who did not have the full set of required tests collected were excluded from the final statistical analysis.

#### 2.1.5. Measures Taken to Reduce Potential Sources of Bias

To address potential sources of bias in this study, participants were selected without considering their age or the presence of erectile dysfunction. Additionally, to avoid measurement bias, the assessment of erectile dysfunction severity was standardized across all patients using the IIEF questionnaire, and laboratory selection was conducted to ensure that all labs used the same type of equipment and calibration methods. Furthermore, to minimize measurement bias, blood samples were collected between 07:00 and 10:00 h. To avoid data collection bias, those assessing erectile function were unaware of the participants’ hormonal levels. Participants were also encouraged to provide honest and accurate information regarding their sexual health and habits, with assurances of confidentiality to reduce the risk of social desirability bias.

Potential confounding factors, such as comorbidities (e.g., diabetes, hypertension), lifestyle factors (e.g., smoking, alcohol consumption), and body mass index (BMI), were not evaluated in this study and may be considered as bias factors.

The study was approved by the Ethics Committee of the Clinical County Hospital Mures, 540136 Târgu Mures, Romania (13756/9 September 2024), Romania, and conducted in accordance with the Declaration of Helsinki

### 2.2. Statistical Analysis

The data were categorized as either nominal or quantitative variables. Nominal variables were described using frequency counts, while quantitative variables were assessed for normality of distribution via the Kolmogorov–Smirnov test. Quantitative data were presented as either the median and range (minimum–maximum) or as the mean and standard deviation (SD), depending on the distribution. The chi-squared test was applied to compare the frequencies of nominal variables. For comparing quantitative variables, the appropriate tests used included the *t*-test, Mann–Whitney test, ANOVA (with Bonferroni correction), or Kruskal–Wallis test (with Dunn’s correction). Pearson’s correlation or Spearman’s rho were used to assess correlations between quantitative variables, as suitable. Statistical significance was considered at *p* < 0.05. Statistical analyses were performed using SPSS for Windows, version 23.0 (SPSS, Inc., Chicago, IL, USA). Multivariate analysis was conducted using linear regression, with the International Index of Erectile Function (IIEF) as the dependent variable and age, FSH, LH, total testosterone (TT), free testosterone (FT), estradiol, prolactin (PRL), and SHBG as independent variables.

## 3. Results

Out of the total of 437 patients who met the inclusion criteria, 411 completed the full IIEF questionnaire and had the entire set of required tests collected and were included in the final statistical analysis.

For variables with a Gaussian distribution (age and IIEF), the descriptive data analysis was performed using the mean and standard deviation (SD), applying the Student’s *t*-test. For variables without a Gaussian distribution (hormonal evaluations), the data were expressed as the median, minimum, and maximum values, with the Mann–Whitney U test applied.

The mean age of the patients was 63.19 years, with an age range between 18 and 88 years. The mean IIEF score in the studied group was 42 (Table 1).

The descriptive analysis of the study group in terms of laboratory data is presented in Table 1.

We evaluated the correlations between age, IIEF, and hormonal values in the studied group. Since most of the variables had a non-Gaussian distribution, Spearman rank correlation coefficient was used to assess these correlations (Table 2).

There is a statistically significant negative correlation between IIEF and age: the older the patient, the greater the degree of erectile dysfunction. This negative correlation is also maintained between IIEF and FSH (*p* < 0.05). A positive significant correlation was observed between IIEF, TT, and FT serum levels in the studied patients: the greater the degree of erectile dysfunction, the lower the testosterone levels (*p* < 0.05).

Age showed a significant correlation with all evaluated hormones (*p* < 0.01), except for estradiol and PRL.

When analyzing the correlations among the laboratory results for the studied hormones, a statistically significant negative correlation was observed between FSH levels and testosterone levels (both total and free), as well as estradiol. Conversely, FSH showed a positive correlation with LH and PRL levels (*p* < 0.05). With the exception of SHBG, TT and FT showed statistically significant correlations with all evaluated hormones.

In our group, no correlation was found between LH and testosterone levels (total and free) (Table 2).

We further evaluated hormonal changes based on the severity of erectile dysfunction. Among the 411 patients assessed, the vast majority (91.73%) exhibited some degree of ED. The mean age of the patients increased with the severity of ED, ranging from 56.26 years for patients with intact erectile function to 73.12 years for those with severe ED (Table 3).

To highlight the changes in hormone levels, we calculated their median values for each degree of erectile dysfunction. FSH, LH, and SHBG showed an increase with each progressive severity of erectile dysfunction. In contrast, both free and total testosterone levels decreased, with the lowest values recorded in patients with severe erectile dysfunction (Table 4).

The ANOVA test, combined with the Bonferroni multiple comparison test, was applied to analyze differences in the mean ages of patients across severity categories based on the interpretation of the IIEF questionnaire. Significant differences were identified, except for the comparison between the mean ages for IIEF scores of 61–75 and the mean ages for IIEF scores of 41–60 (Table 5).

Using multivariate linear regression, we identified that IIEF is significantly associated with age (negative coefficient: higher age corresponds to lower IIEF scores) and with testosterone and free testosterone levels (positive coefficients: lower levels correspond to lower IIEF scores, while higher levels correspond to higher IIEF scores). However, in this regression model, FSH no longer shows a statistically significant association (Table 6).

## 4. Discussion

There is a consensus that age negatively affects erectile function. In our study, a statistically significant correlation was found between age and IIEF, with IIEF scores gradually decreasing as age increased. Among patients with normal erectile function, the mean age was 56.26 years, whereas it increased to 73.12 years in the group with severe erectile dysfunction.

All hormones evaluated in the study, except for estradiol and PRL, demonstrated a statistically significant correlation with age (*p* < 0.05). Total testosterone and free testosterone were negatively correlated with age, while LH, FSH, and SHBG were positively correlated.

Total testosterone levels progressively decreased across each severity group of erectile dysfunction, from a median of 7.05 ng/mL in patients with normal erectile function to a median of 3.56 ng/mL in those with severe symptoms. However, the median values did not fall below the normal minimum threshold (3 ng/mL). Similarly, free testosterone levels showed a progressive decline in median values depending on the severity of erectile dysfunction. The difference was that only the group of patients with normal erectile function had a median within the normal range, whereas the median FT levels for all groups with varying degrees of erectile dysfunction fell below the normal minimum threshold. The statistical analysis (Spearman’s rho and multivariate linear regression) of the relationship between testosterone levels (total and free) and IIEF revealed a statistically significant correlation between these levels and the severity of erectile dysfunction (*p* < 0.05). Additionally, total and free testosterone levels were inversely correlated with age (*p* < 0.05).

In our cohort, there were no statistically significant correlations between PRL, LH, estradiol, SHBG, and erectile dysfunction. Although LH, PRL, and SHBG showed increases in median values across each severity group of erectile dysfunction, and estradiol demonstrated decreases, these changes did not statistically correlate with the severity of erectile dysfunction. Instead, these variations appeared to be more closely associated with age, as previously described. On the other hand, FSH, which also showed an increase in median values with the worsening of erectile function, demonstrated a statistically significant correlation with the severity of erectile dysfunction when using Spearman’s rho correlation between these quantitative variables. However, when applying a multivariate regression model, IIEF was no longer significantly associated with FSH.

The impact of aging on testosterone levels continues to be a topic of ongoing discussion and debate. Some studies have reported normal testosterone levels in healthy older individuals [42,43]. However, most studies have shown that serum TT levels are significantly lower in older men compared to younger men, with a gradual and progressive decline observed over time [44,45,46]. The decrease in FT levels is significantly more substantial. Both the European Male Aging Study (EMAS) [47] and the Baltimore Longitudinal Study of Aging (BLSA) [44] have shown that the age-related decline in FT levels is much greater than the decline in TT levels.

Roelfsema et al. [48] observed that PRL levels are associated with body mass index (BMI) and gender but not with age. Likewise, estradiol levels remained unchanged in aging males [49]. In the same study, Greenblatt et al. [49] found that FSH and LH levels began to increase in men during their 40s, with the rise becoming more pronounced in the subsequent decades of life.

Aribas et al. [50] reported a linear increase in SHBG levels with age. When stratified by age, older participants showed higher SHBG levels [46,51]. Aging-related increases in serum SHBG levels in men may be attributed to enhanced synthesis [52].

The role of testosterone in the pathophysiology of ED has been widely studied, yet the evidence regarding its correlation with ED remains inconclusive and subject to debate. [53]. As men age, serum testosterone levels typically decline. However, in most cases, this decrease is not sufficient to cause ED [54]. Some studies have reported no significant association between testosterone levels and sexual desire or ED [55,56]. Kupelian et al. [57] concluded that there was no correlation between total or bioavailable testosterone levels and ED. However, they observed that men with elevated LH levels combined with higher testosterone levels had a reduced risk of developing ED.

The effects of low TT and FT levels vary. Wu et al. [58] concluded that total and free testosterone levels are closely linked to overall sexual function in middle-aged and older men. Moreover, men with low total and free testosterone levels in the EMAS study [47] were more likely to experience reduced morning erections and ED. They also reported a lower frequency of sexual thoughts compared to those with normal testosterone levels.

As mentioned earlier, there were no statistically significant correlations between PRL, LH, estradiol, SHBG, and erectile dysfunction. Similar to these findings, Weizman et al. [59] reported that no clear correlation was observed between elevated serum PRL levels and ED. Notably, a significant proportion of adult male sex offenders were found to have PRL levels above the normal range [60].

Talha et al. [30] reported a statistically significant correlation between TT and FSH. However, in multivariable analyses, LH, FSH, and PRL levels did not show a significant association with ED [16]. Similarly, Hwang et al. [61], in a study involving 680 patients, found no statistically significant association between the severity of ED and FSH, LH, PRL, or SHBG levels.

The correlation between estradiol levels and the severity of ED is also a topic of debate. Wu et al. [62], in a meta-analysis, demonstrated a significant correlation between estradiol levels and ED. Similarly, Hui-Rong Chen [63] concluded that elevated serum estradiol levels may impair erectile function and play a role in the pathogenesis of organic erectile dysfunction.

Conversely, Castelló-Porcar et al. [64] did not find any association between testosterone/estradiol imbalance and changes in erectile function or sexual desire. However, it seems that, while elevated estradiol levels may exacerbate the condition, low testosterone levels remain the primary factor influencing erectile function [65].

Free testosterone should be considered an essential biochemical marker in the assessment of male hypogonadism, complementing the measurement of total testosterone. Given its critical role in reflecting bioavailable testosterone levels, free testosterone provides valuable insights into a patient’s hormonal status and is crucial for an accurate diagnosis. On the other hand, the evaluation of other markers, such as FSH, LH, prolactin, SHBG, and estradiol, is typically unnecessary in the routine work-up of erectile dysfunction, as elevated levels of these hormones are infrequent and have minimal impact on erectile function. Therefore, focusing on free testosterone alongside total testosterone can streamline the diagnostic process, ensuring a more precise and effective approach to managing male hypogonadism.

The strengths of this study include both the large patient sample size and the correlation between erectile dysfunction, age, and a wide range of hormones (seven) that may have a significant impact on erectile function. A large patient sample provides greater statistical power, which enhances the relevance and validity of the study’s conclusions. Such a sample allows for more accurate data collection that can be more widely generalized to the broader population.

Another important aspect of this study is the analysis of a broad spectrum of hormones that may influence erectile function. By including a wide range of hormones in the analysis, the study has the potential to uncover the complex interactions between these factors, contributing to a deeper understanding of how hormonal imbalances can affect male sexual health.

The study had several limitations. It was a multicenter study where the protocol for evaluating erectile dysfunction and hormonal assessments was standardized, and patient selection was carried out through local advertisements and general practitioners. Although the patients’ ages ranged from 18 to 88 years, the number of patients under 50 years old was small, with only 30 out of the total 411. Therefore, we believe that including a larger number of subjects from these age groups could strengthen the conclusions.

Second, the study did not assess certain conditions, such as metabolic syndrome, obesity, or diabetes, and their correlations with ED, age, or hormonal changes (especially testosterone).

Third, the study did not evaluate the presence of hyperthyroidism or hypothyroidism, considering the possible impact of alterations in normal thyroid values on erectile function.

## 5. Conclusions

Age correlates statistically significantly with both the severity of erectile dysfunction and the levels of total testosterone, free testosterone, LH, FSH, and SHBG. Prolactin and estradiol do not exhibit this correlation. Total and free testosterone are statistically correlated with the severity of erectile dysfunction, showing lower levels as severity increases. However, the median values for total testosterone did not fall below the normal minimum threshold. In the case of free testosterone, all patients with erectile dysfunction had median values above the normal minimum threshold. Free testosterone should be incorporated as a standard biochemical marker, alongside total testosterone, in the diagnostic approach to male hypogonadism. LH, estradiol, SHBG, and prolactin did not show a statistical correlation with erectile dysfunction. Although FSH demonstrated a statistical correlation with erectile dysfunction using Spearman’s rho correlation, this correlation was no longer present when applying a multivariate regression model. The infrequent occurrence and minimal impact of elevated FSH, LH, prolactin, SHBG, or estradiol levels in cases of erectile dysfunction do not support their routine evaluation.

## Figures and Tables

**Table 1:** Descriptive analysis of the study group in terms of hormonal evaluations.

	FSH	LH	Total Testosterone	Free Testosterone	Estradiol	Prolactin	SHBG
			(TT)	(FT)			
Median	6.25	7.02	16.12	13.28	81.32	6.62	38.66
Minimum	0.48	1.67	0.48	1.25	4.25	0.37	6.38
Maximum	94.20	80.00	58.25	382.43	1449.48	105.60	265.12
N	411	411	411	411	411	411	411

**Table 2:** The use of Spearman’s rho to evaluate the relationship between age, IIEF, and hormonal values in the patients included in the study.

			IIEF	Age	FSH	LH	TT	FT	Estradiol	PRL	SHBG
	IIEF	Correlation Coefficient	1.000	−0.502 **	−0.148 **	−0.066	0.338 **	0.427 **	0.047	−0.046	−0.073
Spearman’s rho		Sig. (2-tailed)	-	0.000	0.003	0.181	0.000	0.000	0.341	0.355	0.142
	Age (y)	Correlation Coefficient	−0.502 **	1.000	0.175 **	0.248 **	−0.129 **	−0.130 **	0.030	0.060	0.260 **
		Sig. (2-tailed)	0.000	-	0.000	0.000	0.009	0.008	0.541	0.223	0.000
	FSH	Correlation Coefficient	−0.148 **	0.175 **	1.000	0.502 **	−0.280 **	−0.160 **	−0.175 **	0.190 **	0.015
		Sig. (2-tailed)	0.003	0.000	-	0.000	0.000	0.001	0.000	0.000	0.766
	LH	Correlation Coefficient	−0.066	0.248 **	0.502 **	1.000	−0.167 **	−0.032	−0.108 *	0.325 **	0.312 **
		Sig. (2-tailed)	0.181	0.000	0.000	-	0.001	0.511	0.028	0.000	0.000
	TT	Correlation Coefficient	0.338 **	−0.129 **	−0.280 **	−0.167 **	1.000	0.701 **	0.302 **	−0.150 **	0.079
		Sig. (2-tailed)	0.000	0.009	0.000	0.001	-	0.000	0.000	0.002	0.109
	FT	Correlation Coefficient	0.427 **	−0.130 **	−0.160 **	−0.032	0.701 **	1.000	0.134 **	−0.072	0.056
		Sig. (2-tailed)	0.000	0.008	0.001	0.511	0.000	-	0.007	0.147	0.258
	Estradiol	Correlation Coefficient	0.047	0.030	−0.175 **	−0.108 *	0.302 **	0.134 **	1.000	−0.089	0.169 **
		Sig. (2-tailed)	0.341	0.541	0.000	0.028	0.000	0.007	-	0.071	0.001
	Prolacin	Correlation Coefficient	−0.046	0.060	0.190 **	0.325 **	−0.150 **	−0.072	−0.089	1.000	0.170 **
		Sig. (2-tailed)	0.355	0.223	0.000	0.000	0.002	0.147	0.071	-	0.001
	SHBG	Correlation Coefficient	−0.073	0.260 **	0.015	0.312 **	0.079	0.056	0.169 **	0.170 **	1.000
		Sig. (2-tailed)	0.142	0.000	0.766	0.000	0.109	0.258	0.001	0.001	-
		N	411	411	411	411	411	411	411	411	411

*. The mean difference is significant at the 0.05 level. **. Correlation is significant at the 0.05 level (2-tailed).

**Table 3:** The distribution of patients according to the severity of erectile dysfunction in relation to age.

		Age	IIEF
	Mean	56.26	65
IIEF = 61–75	Std. Deviation	12.70	4
	N (%)	34 (8.27)	34 (8.27)
	Mean	59.92	49
IIEF = 41–60	Std. Deviation	9.58	5
	N (%)	213 (51.83)	213 (8.27)
	Mean	66.36	32
IIEF = 21–40	Std. Deviation	7.84	6
	N (%)	103 (25.06)	103 (8.27)
	Mean	73.16	16
IIEF < 20	Std. Deviation	8.52	1
	N (%)	61 (14.84)	61 (8.27)
Total	N (%)	411 (100)	411

**Table 4:** The distribution of hormone levels across the four categories according to the IIEF.

		FSH	LH	Total Testosterone	Free Testosterone	Estradiol	PRL	SHBG
	Median	5.6	6.3	24.44	37.51	84.59	5.74	35.83
IIEF = 61–75	Minimum	1.54	2.95	4.96	3.43	10.09	0.85	10.61
	Maximum	31.65	25.20	58.25	382.43	1448.95	26.84	265.12
	**N**	34	34	34	34	34	34	34
	Median	5.66	6.61	5.50	23.78	81.66	6.51	37.36
IIEF = 41–60	Minimum	0.48	1.67	19.07	1.98	14.53	0.42	6.38
	Maximum	67.63	43.23	0.49	302.51	489.32	102.40	201.12
	**N**	213	213	50.14	213	213	213	213
	Median	6.81	7.34	13.28	10.96	82.91	7.24	39.740
IIEF = 21–40	Minimum	1.30	2.14	1.46	1.59	4.26	1.13	7.71
	Maximum	40.99	80.00	44.21	358.50	273.78	105.60	143.64
	**N**	103	103	103	103	82.91	103	103
	Median	8.49	8.04	12.34	10.71	75.75	6.63	43.72
IIEF < 20	Minimum	1.56	1.78	0.52	1.25	16.99	0.37	7.95
	Maximum	94.20	74.82	45.42	382.43	308.35	58.31	198.02
	**N**	61	61	61	61	61	61	61
	Median	6.25	7.02	16.12	13.28	81.29	6.62	38.66
	Minimum	0.48	1.67	0.49	1.25	4.26	0.37	6.38
**Total**	Maximum	94.20	80.00	58.25	382.43	1448.95	105.60	265.12
	**N**	411	411	411	411	411	411	411

**Table 5:** The differences in the mean ages of patients across severity categories based on the interpretation of the IIEF questionnaire.

Dependent Variable	(I) IIEF Groups	(J) IIEF Groups	Mean Difference (I–J)	Std. Error	*p* Value	95% Confidence Interval	
						Lower Bound	Upper Bound
Age	IIEF = 61–75	IIEF = 21–40	−10.0945 *	1.84530	0.000	−14.9868	−5.2022
		IIEF = 41–60	−3.6555	1.72300	0.207	−8.2235	0.9126
		IIEF?20	−16.8992 *	1.99675	0.000	−22.1930	−11.6054
	IIEF = 21–40	IIEF = 61–75	10.0945 *	1.84530	0.000	5.2022	14.9868
		IIEF = 41–60	6.4390 *	1.11970	0.000	3.4705	9.4076
		IIEF?20	−6.8047 *	1.50732	0.000	−10.8009	−2.8085
	IIEF = 41–60	IIEF = 61–75	3.6555	1.72300	0.207	−0.9126	8.2235
		IIEF = 21–40	−6.4390 *	1.11970	0.000	−9.4076	−3.4705
		IIEF?20	−13.2437 *	1.35484	0.000	−16.8357	−9.6518
	IIEF < 20	IIEF = 61–75	16.8992 *	1.99675	0.000	11.6054	22.1930
		IIEF = 21–40	6.8047 *	1.50732	0.000	2.8085	10.8009
		IIEF = 41–60	13.2437 *	1.35484	0.000	9.6518	16.8357

Based on observed means. The error term is Mean Square (Error) = 87.043. *. The mean difference is significant at the 0.05 level.

**Table 6:** The multivariate regression model: IIEF is significantly associated with age, testosterone, and free testosterone levels.

Dependent Y		IIEF Multiple Regression		
Independent Variables	Coefficient	Std. Error	*t*	*p*
Age	−0.7071	0.05872	−12.042	<0.0001
FSH	−0.07239	0.07619	−0.950	0.3426
LH	0.07877	0.09799	0.804	0.4220
Testosterone	0.9276	0.2499	3.712	0.0002
Free testosterone	0.1559	0.04564	3.416	0.0007
Estradiol	−0.007864	0.02765	−0.284	0.7762
Prolactin	0.05218	0.06019	0.867	0.3865
SHBG	0.02780	0.01830	1.519	0.1296

## Data Availability

The research data that support the findings are not publicly available. The dataset is available upon request from the authors in accordance with the hospital rules, patients’ consent, and local ethics committee.
